# Notes and News

**Published:** 1896-11-07

**Authors:** 


### Notes and News.

(Collected and Communicated.)

At Alais, in France, a monument has just been erected to Pasteur. The monument consists of a bronze statue of the late scientist, and is placed on a high white marble pedestal. In front kneels a girl in an attitude of supplication. The statue is placed in the Place de la Marechale, and has an effective background of mountains and the ancient fortress of Vaubon.

The Sheppard Lunatic Asylum in the United States has received a munificent bequest of upwards of two million dollars. It is intended to utilise the income accruing from this sum, when invested, to complete the present buildings, and later to provide further accommodation for two hundred patients, and finally to offer free treatment to the indigent insane.

An appeal is being made to establish a seaside holiday and convalescent home for the blind at Southend-upon-Sea. The scheme is directed towards providing accommodation for twelve persons sent by societies and private individuals interested in the welfare of the blind. It is proposed to make a charge of 10s. weekly for each inmate, which will, of course, not cover the expense of maintenance. Funds are asked for. The project is entirely in its infancy. Contributions may be sent to Barclay and Co., bankers, Southend, Essex.

French medical students who are undergoing their year's military service are in future to be distributed especially among those detachments which do not carry an army surgeon. They will be charged with the duty of rendering first aid in accident or emergency pending the arrival of the qualified doctor attached to the military medical service. The students will receive their military instruction along with the companies among which they are distributed. Pharmaceutical and ecclesiastical students will be dealt with in the same way. The object of these measures is to diminish the number of young men who, from being attached to the hospitals and infirmaries, escaped part of their military training.

The treasurer of the Middlesex Hospital has received a donation of £100 from M.H.S.

The house-to-house collection on behalf of the Ipswich Hospital, which has been carried out this year, amounts to upwards of £500.

The Assistance Publique of Paris has received legacies of 10,000 francs from the late Mons. Rau, and of 100,000 through the will of Mons. Travers de Beauvert.

We have been requested to insert the following: The Agent-General for South Australia has received the following telegram from the Government: "Adelaide Hospital crowded; everything satisfactory; you can contradict rumours to the contrary."

At the last meeting of the Metropolitan Asylums Board the Rev. G. W. Pope proposed that a committee should be appointed to consider the desirability of appointing a Fever Hospitals Committee to deal with general principles affecting the managers' hospitals. The motion was, however, defeated by one vote. This is much to be regretted, for, however well the various hospitals may be managed, their separate committees occupy a semi-independent position which is very anomalous, and hardly conducive either to economy or discipline.

The difficulty which has occurred at the Swansea Hospital in regard to the election of a house physician emphasises once more the importance of every hospital having a special committee by whom all such appointments should be made, and of keeping carefully to the rules in the conduct of the business in hand. Any committee to whom the important duty is entrusted of appointing a resident medical officer to a large hospital are not only under an obligation to the sick poor and to the governors, for whom they are trustees, but also to the candidates, who have often put themselves to great trouble in the matter, to conduct the business of selection and finally of election with such care and with such attention to detail, and with such proper record of their proceedings, as to be manifestly fair to all parties. Their actions should also be so precisely in accordance with the rules that they would come out unscathed if perchance the whole proceedings should have to be reviewed by a court of law. We wonder how often this is the case! Clearly in this affair at Swansea the delegation of their duties by the committee to the medical staff had been done in a most informal manner, "to save time," and the final result, when the committee took the work into their own hands, was such as entirely to abolish all confidence in their power to form a decent judgment on the matter. There were ten candidates, and at a meeting held on a Monday these were first by ballot reduced to three, and then by a second ballot arranged in a certain order, the first and the last being men who had already been selected by the medical staff. On the succeeding Thursday another meeting was held to make a final election out of the three selected candidates, when the one whom the same body had on the Monday placed at the head of the poll received not a single vote, while the only one of the three who had not been on the list sent down by the medical staff was elected. What had happened in the meantime? There is certainly something at the back of all this which does not redound to the credit of the management of the Swansea Hospital.

